# TFEB Overexpression in the P301S Model of Tauopathy Mitigates Increased PHF1 Levels and Lipofuscin Puncta and Rescues Memory Deficits^1^,^2^

**DOI:** 10.1523/ENEURO.0042-16.2016

**Published:** 2016-05-23

**Authors:** Hongjie Wang, Ruizhi Wang, Ivan Carrera, Shaohua Xu, Madepalli K. Lakshmana

**Affiliations:** 1 Section of Neurobiology, Torrey Pines Institute for Molecular Studies, Port Saint Lucie, Florida 34987; 2 Department of Neuroscience, Euroespes Biotechnology, 15165A A Coruña, Spain; 3 Florida Institute of Technology, Melbourne, Florida 32901

**Keywords:** Alzheimer’s disease, autophagy, lysosomes, PHF-tau, tauopathy, TFEB

## Abstract

Transcription factor EB (TFEB) was recently shown to be a master regulator of autophagy lysosome pathway. Here, we successfully generated and characterized transgenic mice overexpressing flag-TFEB. Enhanced autophagy in the flag-TFEB transgenic mice was confirmed by an increase in the cellular autophagy markers, as determined by both immunoblots and transmission electron microscopy. Surprisingly, in the flag-TFEB mice we observed increased activity of senescence-associated β-galactosidase by ∼66% of neurons in the cortex (*p* < 0.001) and 73% of neurons in the hippocampus (*p* < 0.001). More importantly, flag-TFEB expression remarkably reduced the levels of paired-helical filament (PHF)-tau from 372% in the P301S model of tauopathy to 171% (*p* < 0.001) in the cortex, and from 436% to 212% (*p* < 0.001) in the hippocampus. Significantly, reduced levels of NeuN in the cortex (38%, *p* < 0.001) and hippocampus (25%, *p* < 0.05) of P301S mice were almost completely restored to WT levels in the P301S/flag-TFEB double-transgenic mice. Also, levels of spinophilin in both the cortex (*p* < 0.001) and hippocampus (*p* < 0.001) were restored almost to WT levels. Most importantly, the age-associated lipofuscin granules, which are generally presumed to be nondegradable, were reduced by 57% (*p* < 0.001) in the cortex and by 55% (*p* < 0.001) in the hippocampus in the double-transgenic mice. Finally, TFEB overexpression in the P301S mice markedly reversed learning deficits in terms of spatial memory (Barnes maze), as well as working and reference memories (T maze). These data suggest that the overexpression of TFEB can reliably enhance autophagy *in vivo*, reduce levels of PHF-tau, and thereby reverse the deposition of lipofuscin granules and memory deficits.

## Significance Statement

The transcription factor EB (TFEB) was recently shown to enhance the entire autophagy lysosome pathway by upregulating >35 genes necessary for lysosome biogenesis and autophagy induction. Surprisingly, we found robustly increased senescence-associated β-galactosidase activity in both the cortex and hippocampus of mice overexpressing flag-TFEB. Interestingly, TFEB expression in the P301S model of tauopathy markedly reduced paired-helical filament-tau levels, which led to significant restoration of synaptic and neuronal markers, as well as learning and memory deficits. More importantly, TFEB expression reduced the accumulation of lipofuscin granules, the first demonstration of its kind *in vivo*. Thus, the activation of TFEB *in vivo* has therapeutic potential not only for tauopathy but for several other neurodegenerative disorders characterized by protein aggregates due to defects in autophagy.

## Introduction

Alzheimer’s disease (AD) and frontotemporal dementia with tau inclusions (FTD-T) are the most common types of dementia (Goedert and Spillantini, 2006). Hyperphosphorylation and aggregation of tau proteins that form aberrant filamentous inclusions that give rise to neurofibrillary tangles are the defining pathological features of tauopathies (Alonso et al., 2001). More than 30 mutations on microtubule-associated protein tau (MAPT) have been reported in patients in whom FTD-T has been diagnosed (Goedert and Jakes, 2005). Although tau mutations do not occur in individuals with AD, increased levels of phosphorylated tau in the CSF correlate with reductions in scores on cognitive tests (Wallin et al., 2006; Mattsson et al., 2009). Moreover, experimental evidence suggests that amyloid-β accumulation precedes and drives tau phosphorylation and aggregation (Götz et al., 2001; Lewis et al., 2001; Oddo et al., 2003). Thus, hyperphosphorylation of tau is also a seminal feature of AD. As of today, there is no effective therapy for tauopathies and AD, and the available treatments can neither reverse nor slow the disease progression, as they are not designed to treat the underlying cause of these diseases. AD has been suggested to have a strong genetic basis with heritability estimates of up to 80% (Gatz et al., 2006). However, the genetic variants in the four well established genes, namely, APP, presenilin (PS) 1, PS2, ApoE, and the newly identified nine genetic risk factors for the late-onset AD altogether account for less than half of this heritability (Kamboh et al., 2012). Therefore, additional risk genes and novel mechanisms that contribute to tauopathies and AD need to be identified.

Importantly, aging is the single most important risk factor for tauopathies and AD, suggesting that there is an age-associated dysfunction of specific molecular and cellular pathways. In fact, accumulating evidence suggests that autophagy, the pathway that involves the delivery of cytoplasmic cargo, including aggregated proteins to the lysosomes, is transcriptionally downregulated during normal aging in the human brain (Martinez-Vicente et al., 2005; Cuervo, 2008; Lipinski et al., 2010), and even more so in AD and tauopathies (Nixon et al., 2005; Nixon, 2007; Ma et al., 2010; Menzies et al., 2015). Compounded with this deficiency, AD and tauopathies have increased the production and aggregation of phosphorylated tau that invariably lead to intracytoplasmic accumulation of protein aggregates. Further, age-related disorders and aging itself are genetically associated with lysosomal dysfunction (Bahr and Bendiske, 2002). Accordingly, the persistent presence of aggregates that leads to irreversible neurodegeneration and clinical symptoms in AD (Cataldo et al., 1996) and other tauopathies (Funderburk et al., 2010) suggests that the autophagy response is either dysfunctional or insufficient.

Recently, it was discovered that the transcription factor EB (TFEB), a basic helix-loop-helix transcription factor, is a master regulator of lysosome biogenesis (Sardiello et al., 2009), which also coordinates autophagy (Settembre et al., 2011), thereby increasing the activity of lysosomal degradative pathways. Moreover, TFEB-induced transcription can stimulate endocytosis (Peña-Llopis et al., 2011) and exocytosis (Medina et al., 2011), which additionally enhance cellular clearance to maintain neuronal proteostasis. Interestingly, TFEB activation has been shown to reduce the accumulation of the pathogenic protein in a cellular model of Huntington’s disease (Sardiello et al., 2009) and a mouse model of Parkinson’s disease (Dehay et al., 2010), which was achieved by gene transfer through viral vectors. This suggests that TFEB-induced lysosome biogenesis can effectively clear protein aggregates in neurons, and may prevent, stop, or even reverse proteinopathy-induced neurodegeneration and associated behavioral deficits. Since autophagy is the sole mechanism of the cell for the bulk degradation of organelles and long-lived proteins (Dunn, 1994; Klionsky and Emr, 2000; Kuma et al., 2004), and since AD and tauopathies are deficient in this function (Cataldo et al., 1996; Funderburk et al., 2010), we were interested to test whether TFEB, as a master regulator of lysosome biogenesis, could ameliorate the pathological hallmarks of paired-helical filament (PHF)-tau in a mouse model.

Here, we generated and characterized a transgenic mouse model overexpressing TFEB in the brain for the first time using bicistronic vector design. Importantly, TFEB overexpression in the P301S model of tauopathy significantly reduced the levels of PHF-tau in the frontal cortex and CA1 region of the hippocampus, which in turn restored neuronal and synaptic markers. Interestingly, TFEB overexpression also significantly reduced the number of lipofuscin puncta in the same brain regions, which led to significant prevention of learning and memory deficits. Thus, the activation of TFEB is a promising strategy to counter dysfunctional autophagy and increased PHF-tau pathology in AD and other tauopathies.

## Materials and Methods

### Chemicals and antibodies

Sudan Black B (SBB; catalog #199664), Nuclear Fast Red (catalog #60700), 5-bromo-4-chloro-3-indolyl β-d-galactopyranoside (catalog #B4252), protease inhibitor cocktail (catalog #P8340), dithiothreitol (catalog #D9779), sodium orthovanadate (catalog #450243), sodium dodecyl sulfate (catalog #L3771), HEPES (catalog #H3375), paraformaldehyde (PFA; catalog #P6148), glutaraldehyde (catalog #G-7776), sodium chloride (catalog #S9888), EDTA (catalog #E9884), potassium ferrocyanide (catalog #P3289), and potassium ferricyanide (catalog #P3667) were all purchased from Sigma-Aldrich. Microcystin-LR (catalog #475815) was purchased from Calbiochem-Millipore. Nonidet P-40 (NP-40) substitute (catalog #M158) was obtained from Amresco. Polyclonal TFEB antibody (catalog #4240) was purchased from Cell Signaling Technology, and monoclonal TFEB antibody (clone S1; catalog #H00007942-M01) was purchased from Abnova. Monoclonal anti-human PHF tau antibody (clone, AT8; catalog #MN1020) was purchased from Thermo Fisher. Monoclonal flag antibody (M2; catalog #F1804) and mouse monoclonal anti-GFP antibody (catalog #SAB5300167) were purchased from Sigma-Aldrich. Anti-Tau antibody, Tau46 (catalog #4019) and anti-microtubule-associated protein 1 light chain 3 alpha (LC3A; catalog #4599) were purchased from Cell Signaling Technology. Rat anti-lysosomal-associated membrane protein 1 (LAMP1) antibody (catalog #1D4B; deposited by J. Thomas August, The Johns Hopkins University School of Medicine, Baltimore, MD) and anti-actin antibody (catalog #JLA20; deposited by Jim Jung-Ching Lin, Cold Spring Harbor Laboratory, Cold Spring Harbor, New York) were purchased from the Developmental Studies Hybridoma Bank (DSHB), University of Iowa (Iowa City, IA). Goat polyclonal cathepsin D antibody (catalog #sc6486) was purchased from Santa Cruz Biotechnology. Secondary antibodies, such as peroxidase-conjugated AffiniPure goat anti-mouse (code #115-035-146), anti-rat (code #112-035-143), anti-sheep (code #313-035-003), and anti-rabbit (code #111-035-144) IgGs were purchased from Jackson ImmunoResearch Laboratories. All antibodies for immunoblot analysis were diluted in 5% nonfat milk in tris-buffered saline with 0.1% Tween-20 buffer.

### Generation of transgenic mice expressing flag-TFEB and enhanced yellow fluorescent protein using bicistronic vector design

The pLenti6.2/V5-TFEB from DNASU (catalog #HsCD00329498) was used as the source of cDNA for human TFEB neuronal isoform E. The TFEB cDNA was amplified by PCR using primers (forward, 5'-aagcttatggcgtcacgcatagggttgcgc-3', and reverse, 5'-gtcgaccagcacatcgccctcctccatgct-3'), and the 1.4 kb of amplified cDNA was cloned into the HindIII and SalI restriction sites in fusion with the 3X flag sequence of p3Xflag-CMV-7.1 plasmid (catalog #E4026, Sigma-Aldrich). To insert viral 2A peptide (P2A) sequence, p3xFlag-TFEB vector was cut with SalI and BamHI and purified. The following two P2A oligos were annealed; P2A-forward, 5'-cggtcgacggaagcggagctactaacttcagcctgctgaagcaggctggagacgtggaggagaaccctggacctggatcccg-3'; and reverse, 5'-cgggatccaggtccagggttct cctccacgtc tccagcctgcttcagcaggctgaagttagtagctccgcttccgtcgaccg-3'. The annealed and purified P2A oligo was cloned in SalI and BamHI sites of p3XFlag-TFEB vector. The resulting plasmid, p3XFlag-TFEB-P2A, was confirmed by sequencing. Using enhanced yellow fluorescent protein (EYFP)-N1 as a template, EYFP was PCR amplified using forward primer 5'-ggatccatggtgagcaagggcgaggagc-3' and reverse primer 5'-ggatccttacttgtacagctcgtccatgcc-3', and was cloned in p3XFlag-TFEB-P2A vector to the BamHI restriction site. The resulting construct was named p3XFlag-TFEB-P2A-EYFP. The lone XhoI site within the TFEB cDNA was removed by site-directed mutagenesis using forward primer 5'-gcaggctcgtgtgcacggcctccctaccacc-3' and reverse primer 5'-ggccgtgcacacgagcctgcatctccagctcc-3'. After confirming the protein expression of both flag-TFEB and EYFP, the entire TFEB-P2A-EYFP cDNA was shuttled and fused with mouse thy-1 promoter at the XhoI restriction site in the pTSC21K plasmid, which was provided by Professor J.W. Gordon (Mount Sinai School of Medicine, New York, NY), which was successfully used in the generation of both RanBP9 (Lakshmana et al., 2012) and COPS5 (Wang et al., 2015) transgenic lines. We used Thy-1 promoter to restrict protein expression only to the postnatal/adult brains to avoid any adverse effects of TFEB during embryogenesis. All cloning was verified by sequencing and protein expression of both flag-TFEB and EYFP, and was confirmed by immunoblots using specific antibodies. The final construct, pTSC21k-flag-TFEB-P2A-EYFP, was linearized by digestion with NotI restriction enzyme, which removed 2349 bp of vector backbone. The microinjection of linearized cDNA in to the blastocyst was performed at the Transgenic Core Facility, Sylvester Comprehensive Cancer Center, University of Miami Health System (Miami, FL) using standard techniques by following animal use protocols as approved by the Institutional Animal Care and Use Committee at the Torrey Pines Institute for Molecular Studies in accordance with National Institutes of Health guidelines. The linearized construct was microinjected into the pronuclei of fertilized C57BL/6 mouse eggs and reimplanted in pseudo-pregnant recipient mice. From 63 pups born from six mothers, genomic DNA was isolated from tails at the time of weaning, and positive mice were identified by genotyping for the transgene. The flag-TFEB-P2A-EYFP-specific primers used in the PCR are as follows: forward primer, 5'-gactacaaagaccatgacggt-3'; and reverse primer, 5'-gtgattgtctttcttctgccg-3', which amplified the expected band of the ∼750 bp fragment. Several founder mice were then backcrossed with native C57BL/6 mice, and colonies were expanded and maintained in the C57BL/6 background.

The B6; C3-Tg (Prnp-MAPT*P301S) PS19Vle/J mice (P301S) were obtained from The Jackson Laboratory (stock #008169) and used as a model of tauopathy. The flag-TFEB mice and P301S mice were bred with each other to generate P301S/flag-TFEB double-transgenic lines. The P301S transgenic mice were identified by genotyping tail DNA using the following primers: forward, 5'-caaatgttgcttgtctggtg-3'; and reverse, 5'-gtcagtcgagtgcacagttt-3'.

### Immunohistochemistry

Immunohistochemical staining was performed in all male wild-type (WT), P301S, and flag-TFEB mouse brains on 20-µm-thick cryostat sections using anti-flag antibody (M2, Sigma-Aldrich) using standard methods (Lakshmana et al., 2012; Wang et al., 2015). Briefly, the primary antibody was incubated overnight, and the immunoreactivity was visualized using Alexa Fluor 568-conjugated anti-mouse secondary antibody for flag. Coverslips were mounted on Vectashield mounting medium with DAPI (Vector Laboratories), and images were obtained using a confocal microscope (C1Si Laser-Scanning Multispectral Confocal Microscope, Nikon).

### Quantitation of proteins in the mouse brains by immunoblotting

Different genotypes of mice were killed with isoflurane and decapitated immediately, and cortical and hippocampal brain regions were rapidly separated and immersed in RIPA buffer containing complete protease inhibitor cocktail together with microcystin and sodium vanadate. Tissue was homogenized using Power Gen 125 (Fisher Scientific) and centrifuged at 100,000 × *g* for 30 min in a Beckman ultracentrifuge. The supernatant was used as the source of SDS-soluble proteins such as PHF-tau, total tau, LAMP1, LC3A, NeuN, spinophilin, and cathepsin D. The rest of the protocol for SDS-PAGE electrophoresis, chemiluminescent detection, and ImageJ quantitation of signals was performed using standard protocols (Lakshmana et al., 2010, 2012; Wang et al., 2015).

### Transmission electron microscopy

Male WT or flag-TFEB transgenic mice were perfused with 4% PFA plus 2.5% glutaraldehyde prepared in 0.1 m sodium phosphate buffer at pH 7.2, and several pieces of brain tissues from cortex or hippocampus (2-3 mm on each side and 0.5 mm thick) were incubated overnight in the same perfusion solution. The brain tissues were postfixed in 1% osmium tetroxide in phosphate buffer for 2-3 h at 4^°^C. After thoroughly rinsing the tissues in phosphate buffer and distilled water, the tissues were dehydrated in 30%, 50%, 75%, and 95% acetone. Dehydration was further continued in 100% fresh acetone and then treated with 100% propylene oxide for 15 min. The tissues were then infiltrated in a mixture of 70% propylene oxide and 30% LX112 resin (catalog #21310, Ladd Research Industries) for 1 h, followed by 50:50 ratio for 1 h and then in 100% embedding mix on a tissue rotator. The samples were frozen at −20^°^C overnight, changed the tissues in a freshly prepared embedding mix and then polymerized them in gelatin capsules (catalog #70100, Electron Microscopy Sciences) at 70^°^C for 2 d. Ultrathin sections of ∼80 nm were cut by an ultramicrotome (EM UC6, Leica) using a diamond knife (DiATOME) and directly placed on nickel grids (catalog #G200-Ni, Electron Microscopy Sciences) coated with parlodian. The sections were stained in 8% uranyl acetate for 30 min followed by lead citrate for 10 min for examination on a Morgagni Transmission Electron Microscope (FEI) operated at 60 kV. Photographs were obtained with an Olympus MegaView III camera (ResAlta Research Technologies) or an ActiveVu 16.8 megapixel bottom-mount camera (AMT Corporation) mounted on the transmission electron microscope.

### β-Galactosidase histochemistry

Male WT and flag-TFEB mice were killed by isoflurane, and 15 μm coronal brain sections were cut in a cryostat and mounted onto glass slides. Sections were rehydrated twice with rinse buffer containing 100 mm sodium phosphate, 2 mm MgCl_2_, and 0.01% sodium deoxycholate, pH 7.3. To stain β-galactosidase (β-gal), sections were immersed in a solution containing 1 mg/ml X-gal, 100 mm sodium deoxycholate, 0.02% NP-40, 5 mm potassium ferricyanide, and 5 mm potassium ferrocyanide, pH 6.0. Sections were then incubated at 37°C for 18 h, washed in PBS, counterstained with 0.1% Nuclear Fast Red, and visualized in a bright-field microscope. β-gal-positive neurons were identified by their intense blue color, and were quantified in the frontal cortical area and CA1 region of the hippocampus in a defined area of 600 µm^2^. The data were analyzed in an unbiased manner by a person blinded to the genotype of the samples. The senescence-associated β-gal (SA-β-gal)-positive neurons were expressed as a percentage of total neurons within the defined area. A total of six sections per mouse, five mice per genotype were used for the quantification.

### Lipofuscin staining by Sudan Black B

To stain lipofuscin, we used a combination of methods published by Gatenby and Moussa (1949) and Rasmussen (1961). In brief, male WT, P301S, and P301S/flag-TFEB mice were killed by isoflurane; brains were rapidly removed and frozen in OCT. The 15 μm cryostat sections were mounted onto Superfrost slides and fixed in 4% PFA. After washing in PBS three times, sections were incubated in 50% and 70% alcohol for 5 min each. An SBB solution was prepared by dissolving 0.7 g in 100 ml of 70% ethanol and stirring overnight. The solution was filtered through filter paper. Care was taken throughout the process to avoid precipitation of the stain by ethanol evaporation. After the dehydration step, a drop of freshly prepared SBB solution was placed on a clean slide, and the tissue sections were placed facing down on the SBB solution for 8 min. The sections were then embedded in 50% ethanol for 3 min, transferred, and washed in distilled water. The sections were also counterstained with 0.1% Nuclear Fast Red for 10 min and mounted in 40% glycerol/TBS mounting medium. The lipofuscin granules were identified as blue-black puncta in the perinuclear and cytoplasmic regions of neurons. The number of puncta were counted within a defined region of frontal cortex and CA1 region of the hippocampus, quantified and compared between the P301S and P301S/flag-TFEB genotypes of mice. The counting and analysis of lipofuscin data were performed by an investigator blinded to the genotype of the mice being analyzed.

### Learning and memory tests by Barnes and T mazes

In the Barnes maze testing, distinct spatial cues were located all around the wall surrounding the maze, which was kept constant throughout the study. The three genotypes of mice tested included male WT, P301S, and P301S/flag-TFEB, all of which were 7 months of age. After habituation of each mouse to the Barnes maze, training was given for 4 d. On each day of the training, the procedure consisted of placing the mouse in the escape box and leaving it there for 1 min. One minute later, the first session started. At the beginning of each session, the mouse was placed in the middle of the maze in a 10-cm-high cylindrical black start chamber. After 30 s, the start chamber was removed, a buzzer (75 dB) and a light (400 lux) were turned on, and the mouse was set free to explore the maze. The session ended when the mouse entered the target quadrant with escape tunnel or after 3 min had elapsed. When the mouse entered the escape tunnel, the buzzer was turned off, and the mouse was allowed to remain in the dark for 1 min. The tunnel is always located underneath the same hole (stable within the spatial environment), which is randomly determined for each mouse. Mice were tested once a day for 4 d, six trials per day per mice for the acquisition portion of the study. For the probe test, which was conducted 72 h after the training period, the escape tunnel was removed and the mouse was allowed to freely explore the maze for 3 min. The time spent in each quadrant was determined, and the total time spent in the target quadrant (the one originally containing the escape box) was compared with the average time spent in the other quadrants. This is a direct test of spatial memory, as there is no potential for local cues to be used in the behavioral decisions of the mouse.

To evaluate learning skills in the T maze paradigm, all three genotypes of mice (i.e., WT, P301S, and P301S/flag-TFEB) at 7 months of age were deprived of food for ∼12 h prior to testing. As a reward, a small food pellet was placed in the left or right arm, which was alternatively designated as the bait arm. To begin the training session, each mouse was initially habituated to the T maze by allowing it to explore the maze for 5 min with all the guillotine doors open. The session consisted of placing the mice in the start box for 30 s and opening the door, and the time to enter the right or left arm was noted. The probe test was conducted 72 h after acquisition for 5 d, 10 trials/d for each mouse. The correct responses (as a percentage) and latency (in seconds) were calculated for each genotype and analyzed.

### Statistical analysis

Immunoblot signals for all proteins in the mouse brains were quantified using publicly available Java-based ImageJ software. Statistical significance was established by either Student’s *t*-test or one-way ANOVA followed by Student–Newman–Keuls *post hoc* test for the pairwise comparison. We used two-tailed *p* values, assuming that populations may have different SEs. The learning and memory skills were analyzed by one-way ANOVA followed by Tukey–Kramer multiple-comparisons *post hoc* test for comparisons among different genotypes of mice using Instat3 software (GraphPad). The data are presented as the mean ± SEM. The data were considered significant only at *p* < 0.05 (* indicates *p* < 0.05, ** or $$ indicates *p* < 0.01, and *** or $$$ indicates *p* < 0.001).

## Results

### Characterization of flag-TFEB transgenic mice

**Figure 1.**
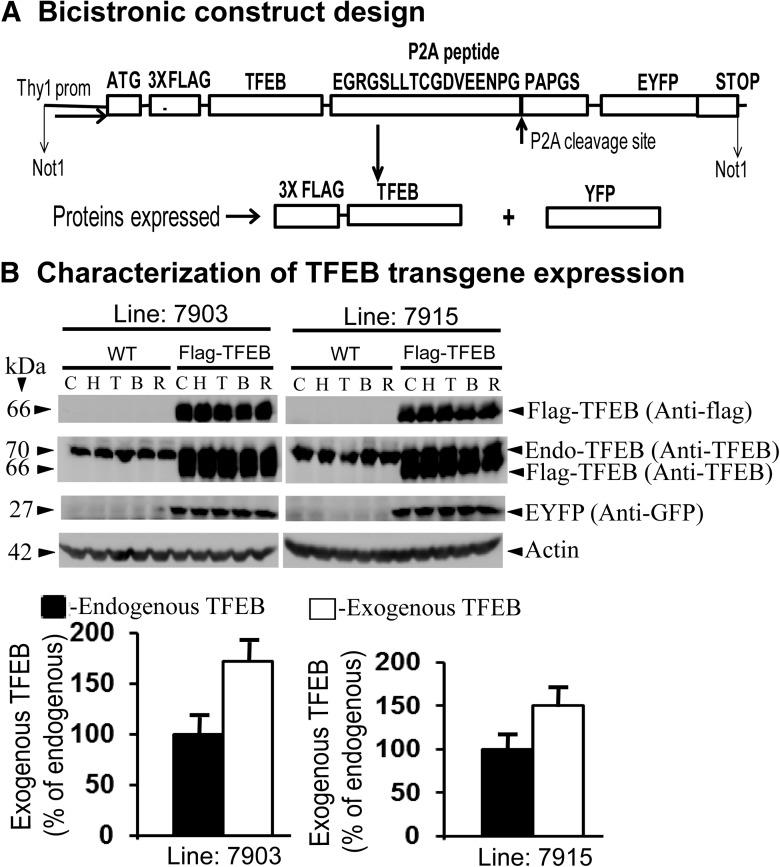
Generation and characterization of transgenic mice overexpressing flag-TFEB and EYFP in the brain. ***A***, Schematic diagram of the bicistronic vector design used in the generation of transgenic mice. PCR-amplified cDNA of the human brain-specific TFEB isoform E was fused in frame with a 3X flag sequence and was followed by the fusion of cDNA encoding P2A and EYFP, as shown. The transgenes were driven by the thy-1 promoter, which restricts protein expression only to the CNS and only during the postnatal period. ***B***, Immunoblots from WT mice or derived from two lines of transgenic mice showing the expression of flag-TFEB in the cortex (C), hippocampus (H), thalamus (T), brainstem (B), and cerebellum (R). Exogenous flag-TFEB was detected by flag antibody (M2), and both endogenous and exogenous TFEB were detected by anti-TFEB antibody. The detection of EYFP by anti-GFP antibody demonstrates the successful use of bicistronic vector. Actin was detected as a loading control. The quantitation of expression levels of exogenous flag-TFEB protein showed a >1.5-fold expression relative to endogenous TFEB levels in both the transgenic lines. The data are reported as the mean ± SEM. *n* = 5 per mouse genotype.

To assess whether the activation of autophagy through the overexpression of TFEB in the brain has any effect on the levels of PHF-tau and its associated neuropathology, we generated transgenic mice overexpressing flag-TFEB in the brain. Because the expression of EYFP in neurons has several advantages, especially as a reporter of transgene expression, we also wanted to coexpress EYFP in the same neurons overexpressing flag-TFEB. Therefore, we used a bicistronic vector design, as shown schematically in Figure 1*A*, and used a viral P2A self-processing strategy (Tang et al., 2009). Since both flag-TFEB and EYFP transgenes are driven by a single promoter and are derived from the same transcript, individual neurons are expected to express both the proteins. We used Thy-1 promoter to drive the transgenes only in the adult brain to avoid unwanted effects of transgenes during development. Also, the use of the flag tag ensured the detection of exogenous flag-TFEB and differentiated it from the endogenous TFEB.

Initial characterization of flag-TFEB transgenic mouse brains with routine hematoxylin and eosin staining revealed no noticeable abnormalities in the cytoarchitecture of brain regions when compared to WT mice. Both body and brain weights of flag-TFEB mice also did not differ from those of the WT controls. Additionally, no behavioral abnormalities of any kind were noticed in the flag-TFEB mice. This ensured that the use of the P2A strategy for the expression of two proteins is feasible and safe. Next, we characterized two lines of flag-TFEB transgenic mice derived from two founder lines, 7903 and 7915, for protein expression by Western blots. Immunoblotting of brain tissues from line 7903 with flag antibody (M2) showed more protein in the cortex and hippocampus, followed by the thalamus, brainstem, and cerebellum. In the line 7915, expression levels were almost equal in all the brain regions (Fig. 1*B*, top panels). Such a differential transgene expression levels in different founder lines is probably integration-site dependent. Flag antibody did not detect any signals in WT brains, suggesting that the signals seen in the flag-TFEB mice are specific to flag-TFEB fusion protein. The TFEB-specific polyclonal antibody was used to detect both the exogenously expressed flag-TFEB and the endogenous TFEB. Because the endogenous mouse TFEB is larger (70 kDa) than the exogenously expressed flag-tagged human TFEB neuronal isoform E (66 kDa), polyclonal TFEB antibody detected flag-TFEB, which ran lower than the endogenous TFEB in the transgenic mice (Fig. 1*B*, Lines 7903 and 7915, top panel), while the WT mice showed only endogenous TFEB (Fig. 1*B*, Lines 7903 and 7915, top panel). Thus, both of the lines of TFEB transgenic mice express flag-TFEB protein, though there are differences among the two lines in regional expression. Quantitation of exogenously expressed flag-TFEB protein levels revealed a difference of >1.5-fold in both lines 7903 and 7915 relative to the endogenous mouse TFEB protein levels (Fig. 1*B*). Importantly, because of the use of a bicistronic strategy, P2A-mediated EYFP protein was also detected in both lines of mice (Fig. 1*B*, bottom middle panel).

**Figure 2.**
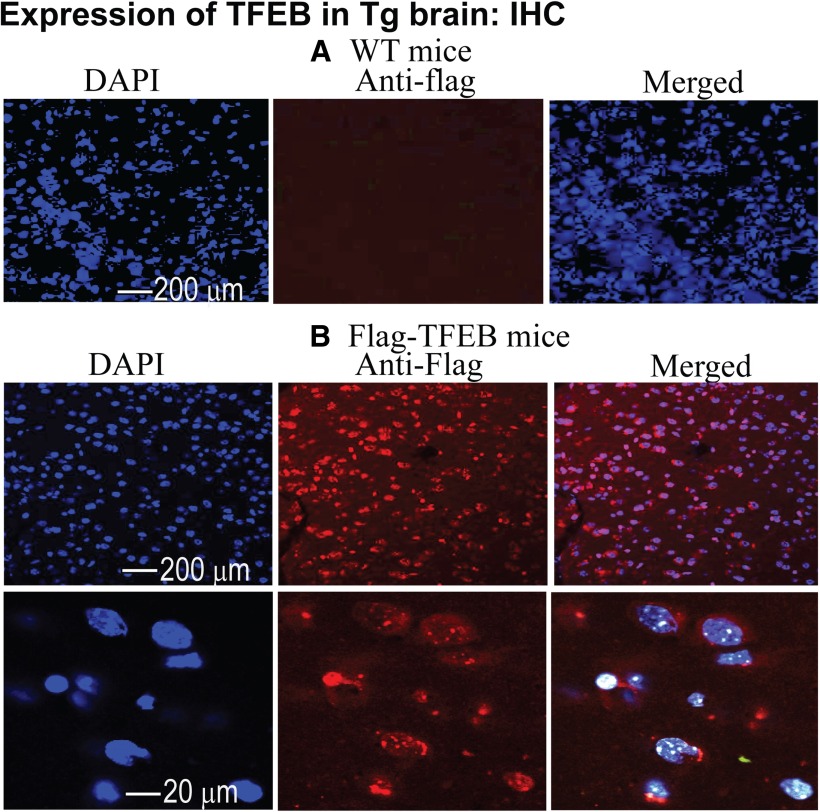
Immunohistochemical demonstration of widespread expression of flag-TFEB in the cortex by confocal microscopy in the transgenic line 7903. ***A***, Cortical brain sections from WT controls showed no signals for flag-TFEB (red, M2 antibody) but showed only DAPI-stained nuclei (blue). ***B***, Brain sections from the transgenic line 7903 shows the expression of flag-TFEB (red). The merged images showed the expression of flag-TFEB both in the nuclei and the cytoplasm.

We also stained flag-TFEB transgenic mouse brains by immunohistochemistry (IHC). Images were acquired in a confocal microscope (C1Si Laser-Scanning Multispectral Microscope, Nikon). Consistent with immunoblotting results, IHC staining with M2 antibody for flag-TFEB demonstrated strong and uniform neuronal staining of flag-TFEB (red, column 2) in the cortex (Fig. 2*B*), whereas in the WT mouse brains only DAPI-stained blue nuclei were detected (Fig. 2*A*), suggesting that fluorescent signals observed with flag antibody (M2) are specific to the flag-TFEB transgene. Omission of either the primary or secondary antibody yielded no staining (data not shown). Thus, immunoblotting and IHC results are consistent with each other and suggest that these transgenic lines are reliable models for studying the effect of overexpression of flag-TFEB in the brain.

### Confirmation of increased autophagy markers in the flag-TFEB mice by transmission electron microscopy

**Figure 3.**
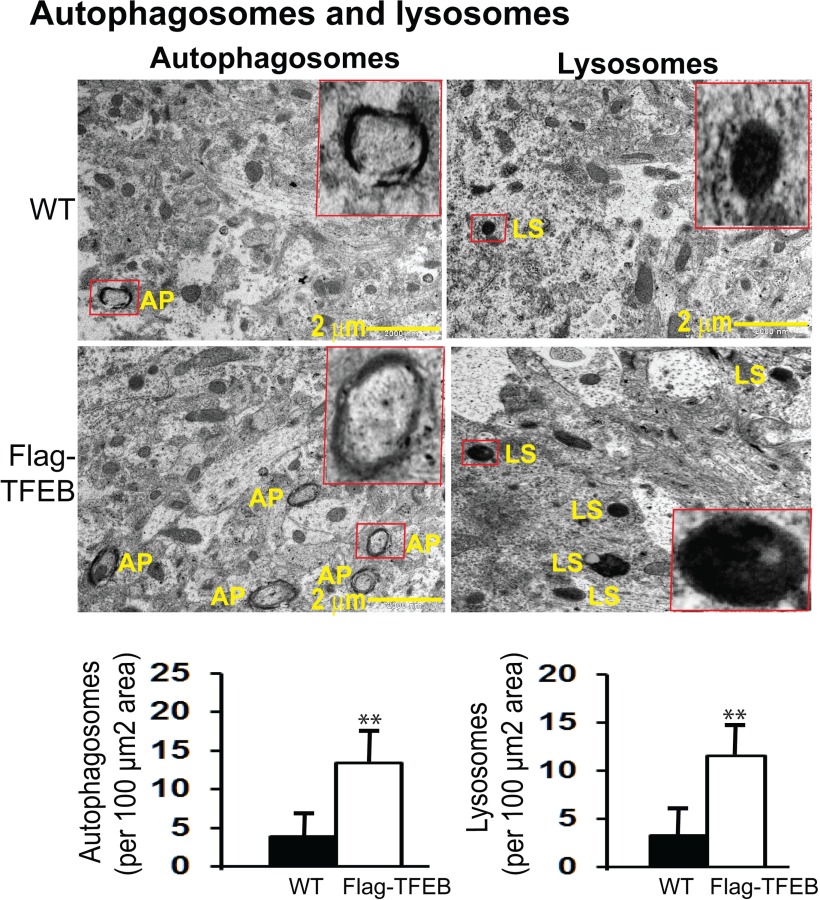
TEM demonstration of increased autophagy markers in the flag-TFEB transgenic mice. The ultramicrotome sections were stained for TEM analysis. Unbiased quantitation showed increases in double-membrane autophagosomes (APs) by 245% per 100 µm^2^ area and in electron-dense LSs by 249% per 100 µm^2^ area in the cortices of flag-TFEB mice compared with those of WT control mice. For each of the autophagy markers, the boxed structures in red are shown at higher magnification for clarity. Data are reported as mean + SEM, n = 4 mice per genotype. **p <0.01 by Student's *t*-test.

**Figure 4.**
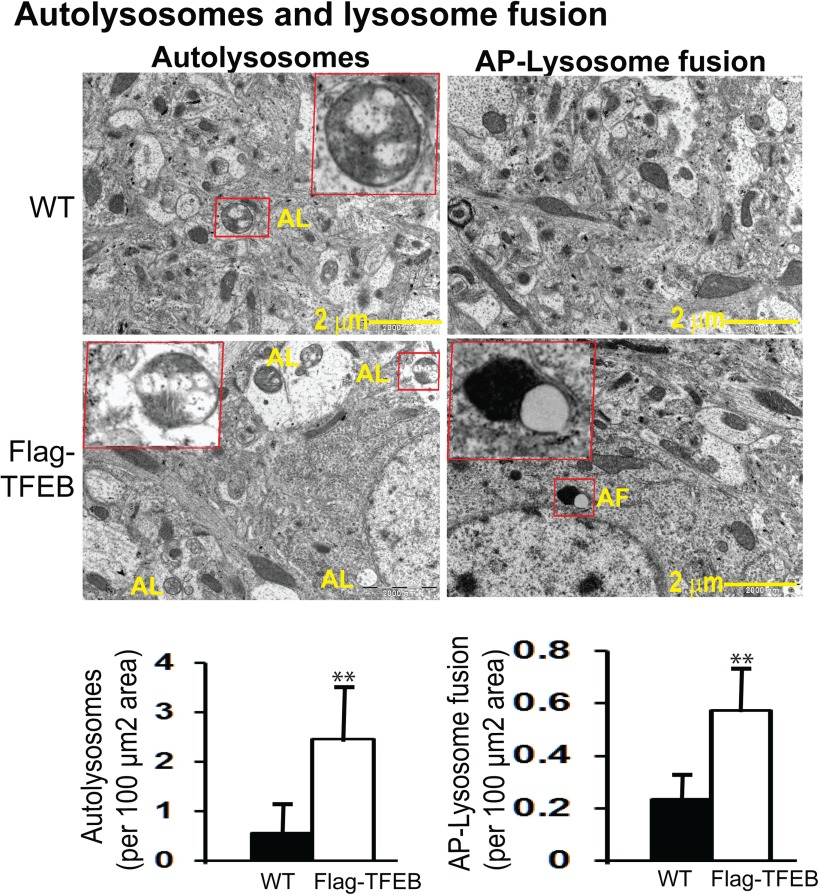
TEM demonstration of increased autophagy markers in the flag-TFEB transgenic mice. The ultramicrotome sections were stained for TEM analysis. Unbiased quantitation also showed increases in single-membrane autolysosomes (AL) by 339% per 100 µm^2^ area and in autophagosome fusion with lysosomes (AF) by 144% per 100 µm^2^ area in the cortices of flag-TFEB mice compared with those of WT control mice. For each of the autophagy markers, the boxed structures in red are shown at higher magnification for clarity. Data are reported as the mean ± SEM. *n* = 4 mice per genotype. **p < 0.01 by Student’s *t*-test.

Since TFEB is known to enhance autophagy, we first wanted to confirm whether autophagy markers are increased in the flag-TFEB brains. Due to its high resolution, electron microscopy remains a gold standard for the accurate detection of autophagy, and therefore we used transmission electron microscopy (TEM) to directly visualize and detect morphological evidence of autophagy (Ylä-Anttila et al., 2009). The autophagosomes (APs) were recognized by their size (0.5-1.5 µm), the presence of undegraded cytoplasm, and, most importantly, a double membrane. Lysosomes (LS) were recognized by their size (0.1-1.0 μm) and their electron-dense appearance. Autolysosomes were recognized by their size (0.5-1.5 μm), the presence of focally degrading cytoplasm, and the presence of a clear single membrane. For unbiased quantitation, a uniform random-sampling method was applied, and all images were acquired at the same magnification, thereby giving every cell profile an equal probability of being included in the counting. In the WT mice, the number of APs detected were ∼3.9/100 µm^2^ area, whereas in the flag-TFEB mice the numbers increased (245%, *p* < 0.01) to 13.45/100 µm^2^ area (Fig. 3, left panels). Similarly, the number of LSs increased from an area of 3.31/100 µm^2^ in the WT mice to 11.55/100 µm^2^ (249%, *p* < 0.01) in the flag-TFEB mice (Fig. 3, right panels). The number of autolysosomes also increased from 0.56 in the vehicle group to 2.46 in the flag-TFEB mice (339%, *p* < 0.01) compared with WT controls (Fig. 4, left panels). Although lysosomes fusing with autophagosomes were rarely seen, quantitation of their small numbers also showed a significant increase in area from 0.235 to 0.574/100 µm^2^ (144%, *p* < 0.01) in the flag-TFEB mice (Fig. 4, right panels). Thus, the increased numbers of autophagosomes, lysosomes, autolysosomes, and lysosomes fusing with autophagosomes are confirmatory evidence that flag-TFEB overexpression activates autophagy in the mouse brain.

### Overexpression of flag-TFEB increases β-galactosidase-positive neurons

**Figure 5.**
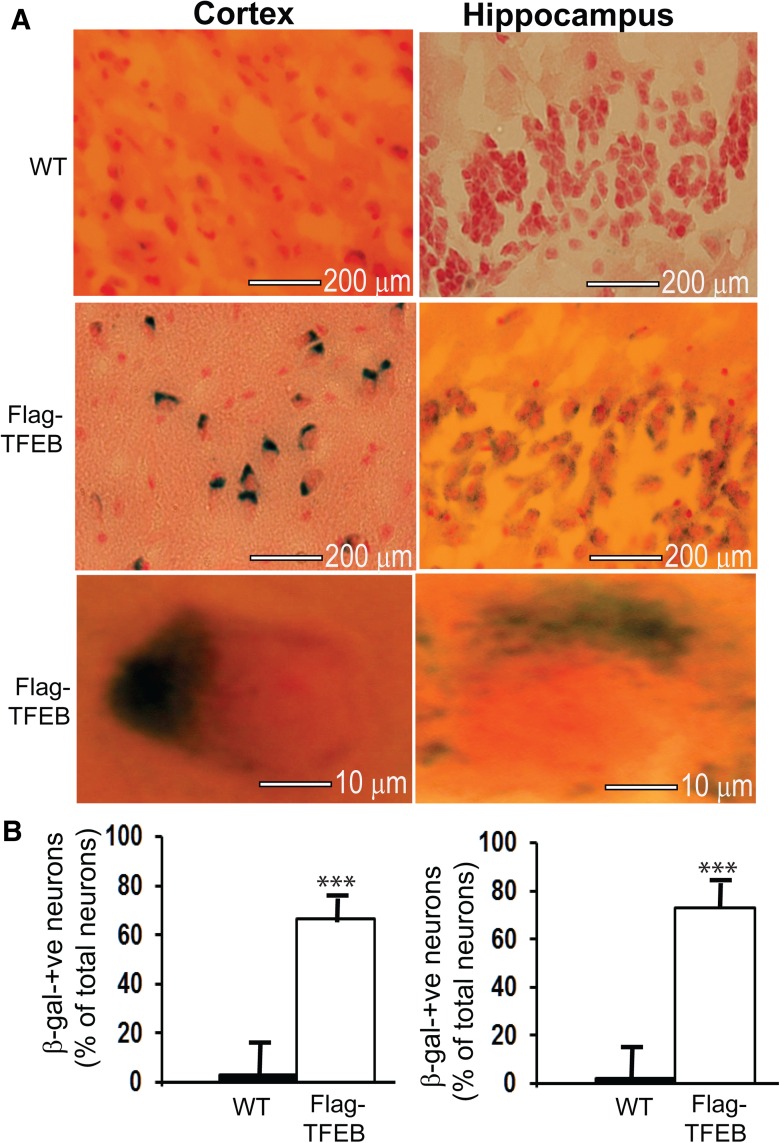
TFEB overexpression robustly increases SA-β-gal activity at 7 months of age. ***A***, Coronal brain sections from WT and flag-TFEB transgenic lines were stained with X-gal at a 1 mg/ml concentration for 18 h at 37°C and then were counterstained with 0.1% Nuclear Fast Red to visualize all neurons. ***B***, SA-β-gal-positive neurons were identified by their intense blue color and were quantified within a defined area of 600 µm^2^ by an investigator who was blind to the genotype of the samples. Six sections per mice, and five mice per genotype were used for the quantification. An average of only 3% of total neurons was positive for SA-β-gal in the frontal cortical areas of WT mice, but in the flag-TFEB mice, 66% of total neurons showed the staining. In the CA1 region of the hippocampus, the WT brains showed only 2% of total neurons to be positive for SA-β-gal, whereas the flag-TFEB mice showed 73% of total neurons to be positive for SA-β-gal. Statistical analysis by *t* test revealed significant differences. ***p < 0.001. The data are reported as the mean ± SEM. *n* = 5 per genotype.

TFEB overexpression is known to stimulate lysosome biogenesis and autophagy (Sardiello et al., 2009; Settembre et al., 2011). Since autophagy has been suggested to promote neuronal health and has been shown to be inversely related to cellular senescence (Kang et al., 2011; Fujii et al., 2012; Wang et al., 2012), and also since the activation of autophagy has been shown to prolong lifespan in different organisms (Simonsen et al., 2008; Eisenberg et al., 2009; Morselli et al., 2010), including mice (Pyo et al., 2013), we wanted to assess whether autophagy activation by long-term overexpression of TFEB has any influence on the markers of cellular senescence. Therefore, we used a widely used SA-β-gal activity (Dimri et al., 1995; Ozcelik et al., 2013) as a marker for neuronal senescence. Contrary to our expectation, the overexpression of flag-TFEB in the transgenic mice robustly increased SA-β-gal-positive neurons in both the frontal cortical area and CA1 region of the hippocampus at 7 months of age (Fig. 5*A*). The SA-β-gal-positive neurons were expressed as a percentage of total neurons within the defined area of 600 µm^2^. Thus, in the cortex of WT mice, we could barely observe any SA-β-gal-positive neurons (only ∼3%), whereas in the TFEB transgenic brains we found nearly 66% (*p* < 0.001) of cells intensely stained for SA-β-gal (Fig. 5*B*). Similarly, we found only 2% of cells showing SA-β-gal staining in the CA1 region of the hippocampus in the WT mice, whereas in the flag-TFEB mice 73% (*p* < 0.001) of neurons showed positive staining (Fig. 5*B*). Thus, there is a robust increase in the number of neurons staining positive for SA-β-gal in the flag-TFEB mice.

### TFEB overexpression reduces PHF-tau levels in the P301S model of tauopathy

**Figure 6.**
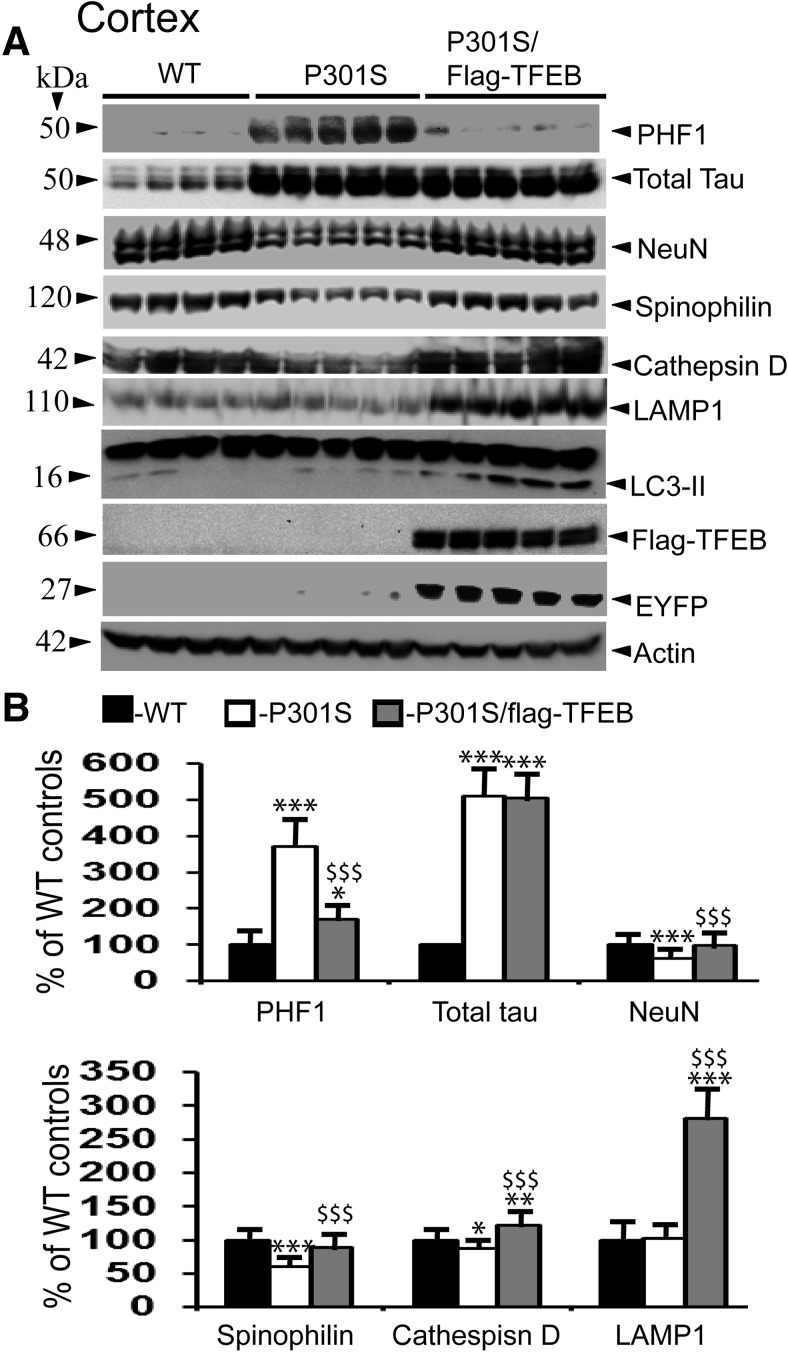
TFEB expression reduces PHF1 levels in the P301S model of tauopathy, and significantly attenuates the loss of neuronal and synaptic markers in the cortex. ***A***, Lysates were prepared from the cortical regions of WT, P301S, and P301S/flag-TFEB mice, and were subjected to immunoblotting. ***B***, Quantitation by ImageJ revealed PHF1 levels of 371% in the P301S mice compared with WT controls, which were significantly attenuated to 171% in the P301S/flag-TFEB double-transgenic mice. However, TFEB expression did not alter the levels of total tau. NeuN levels were reduced by 38% in the P301S mice, which were significantly mitigated to 1% in the double-transgenic mice. Similarly, spinophilin levels were reversed from 40% reduction to 12% due to TFEB overexpression. TFEB expression also increased LAMP1 levels by 181% and cathepsin D levels by 22% in the P301S/flag-TFEB mice compared with WT controls. Expression of flag-TFEB and EYFP were also confirmed. Statistical analysis by one-way ANOVA followed by Student–Newman–Keuls *post hoc* test revealed significant differences. *p < 0.05, **p < 0.01, and ***p < 0.001, compared with WT controls. $$*p* < 0.01, $$$*p* < 0.001, compared with P301S mice. The data are reported as the mean ± SEM. *n* = 4 WT mice; *n* = 5 P301S and P301S/flag-TFEB mice.

**Figure 7.**
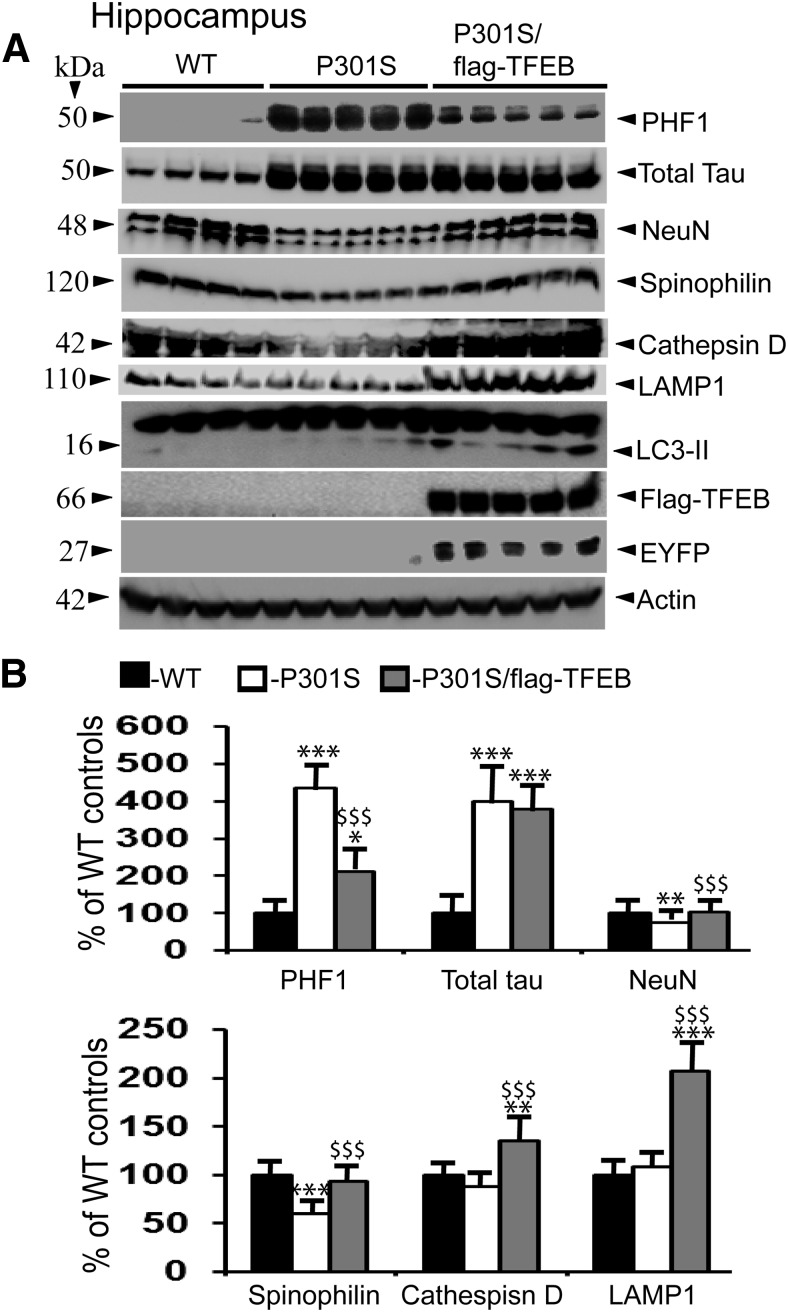
TFEB expression reduces PHF1 levels in the P301S model of tauopathy, and significantly attenuates the loss of neuronal and synaptic markers in the hippocampus. ***A***, Lysates were prepared from the hippocampal regions of WT, P301S, and P301S/flag-TFEB mice, and were subjected to immunoblotting. ***B***, Quantitation by ImageJ revealed PHF1 levels of 436% in the P301S mice compared with WT controls, which were significantly attenuated to 212% in the P301S/flag-TFEB double-transgenic mice. However, TFEB expression did not alter the levels of total tau, which remained similar in the P301S and the double-transgenic mice. NeuN levels were reduced by 25% in the P301S mice, which was significantly reversed to WT levels in the double-transgenic mice. Similarly, spinophilin levels were reversed from 40% reduction to 6% due to TFEB overexpression. TFEB expression also increased LAMP1 levels by 207% and cathepsin D levels by 33% in the P301S/flag-TFEB mice compared with WT controls. Statistical analysis by one-way ANOVA followed by Student–Newman–Keuls *post hoc* test revealed significant differences. *p < 0.05, **p < 0.01, and ***p < 0.001, compared with WT controls; $$*p* < 0.01, $$$*p* < 0.001, compared with P301S mice. The data are reported as the mean ± SEM. *n* = 4 WT mice; *n* = 5 P301S and P301S/flag-TFEB mice.

Since autophagy inducers, such as rapamycin (Ozcelik et al., 2013), trehalose (Schaeffer et al., 2012), and methylene blue (Congdon et al., 2012), have been shown to reduce levels of phosphorylated-tau, which leads to enhanced neuronal survival, we were interested to test whether TFEB-mediated autophagy activation has similar effects *in vivo*. The P301S transgenic model expressing the P301S mutant form of human MAPT, driven by mouse Prnp (prion protein promoter), exhibits many characteristics of human tauopathy, including neuronal tau inclusions and neurodegeneration (Allen et al., 2002). Therefore, we bred flag-TFEB mice with P301S mice and generated a double-transgenic line, P301S/flag-TFEB. Quantitation of total tau levels at 7 months of age did not reveal any appreciable differences between P301S and P301S/flag-TFEB genotypes, in both the cortex (Fig. 6*A*, panel 2, *B*) and the hippocampus (Fig. 7*A*, panel 2, *B*). However, the levels of PHF-tau were reduced from 372% in the P301S model of tauopathy to 171% (*p* < 0.001) when flag-TFEB was overexpressed in the P301S/flag-TFEB double-transgenic mice in the cortex at 7 months of age (Fig. 6*A*, panel 1, *B*). Similarly, PHF-tau levels were markedly reduced from 436% to 212% (*p* < 0.001) in the hippocampus when flag-TFEB was overexpressed in the P301S mice (Fig. 7*A*, panel 2, *B*). These results clearly suggest that TFEB overexpression selectively clears hyperphosphorylated tau species but not the nonphosphorylated forms of tau.

### TFEB expression rescues loss of neuronal and synaptic markers

Since the P301S mouse model of tauopathy exhibits several pathological features of human disease, including neurodegeneration (Hampton et al., 2010), loss of synapses (Yoshiyama et al., 2007), defects in synaptic transmission (Yang et al., 2015), loss of dendritic spines and cortical plasticity (Hoffmann et al., 2013), and aberrant neuronal morphology (Mellone et al., 2013), we were also interested to examine whether a reduction in PHF1 levels by TFEB would mitigate markers of neurodegeneration and synapses. Therefore, we quantified levels of NeuN (neuron marker) and spinophilin (dendritic spine marker). At 7 months of age, the P301S mouse model showed a 38% (*p* < 0.001) reduction in the levels of NeuN in the cortex compared with WT controls, which was remarkably attenuated to only a 1% reduction (*p* < 0.001) in the P301S/flag-TFEB double-transgenic mice (Fig. 6*A*, panel 3, *B*). In the hippocampus, the reduction was only 25% (*p* < 0.05) in the P301S mouse model, which was also attenuated to only 3% in the double-transgenic lines (Fig. 7*A*, panel 3, *B*). Further, spinophilin levels in the cortex were reduced by 40% (*p* < 0.001) in the P301S model, which were significantly alleviated to 12% (*p* < 0.001) in the double-transgenic lines due to TFEB overexpression (Fig. 6*A*, panel 4, *B*). In the hippocampus, the reduction was 40% (*p* < 0.001) in the P301S mouse model when compared with WT controls, which was also significantly reversed to only 6% reduction (*p* < 0.001) in the double-transgenic lines (Fig. 7*A*, panel 4, *B*). Thus, TFEB-mediated reduced PHF1 levels might account for the recovery of both NeuN and spinophilin in these brain regions.

Next, to confirm whether TFEB expression in the P301S mice leads to the biogenesis of lysosomes and autophagy *in vivo*, we quantified protein levels of three lysosomal marker proteins, the LAMP1, a lysosomal membrane-associated protein (Saftig and Klumperman, 2009), LC3-II, a marker of autophagosomes (Kabeya et al., 2000), and cathepsin D, a lysosomal enzyme that mediates the degradation of proteins. Consistent with cell culture studies (Dehay et al., 2010; Spampanato et al., 2013; Kilpatrick et al., 2015), protein levels of both LAMP1 and cathepsin D (Figs. 6*A*,*B*, 7*A*,*B*) were increased in the brain, suggesting that TFEB overexpression in the mouse brain reliably activates autophagy. In the cortex, LAMP1 protein levels were increased by 181% (*p* < 0.001) in the double-transgenic mice compared with WT controls (Fig. 6*A*, panel 6, *B*), while they were increased by 207% (*p* < 0.001) in the hippocampus (Fig. 7*A*, panel 6, *B*). Similarly, cathepsin D levels were increased by 22% (*p* < 0.01) in the cortex (Fig. 6*A*, panel 5, *B*), and by 36% (*p* < 0.01) in the hippocampus (Fig. 7*A*, panel 5, *B*). These results are consistent with TEM data and confirm that TFEB overexpression in the brain increases the number of lysosomes, which may directly account for the reduced PHF1 levels followed by reversal of synaptic and neuronal markers.

### TFEB overexpression significantly reduces lipofuscin puncta in the P301S model of tauopathy

**Figure 8.**
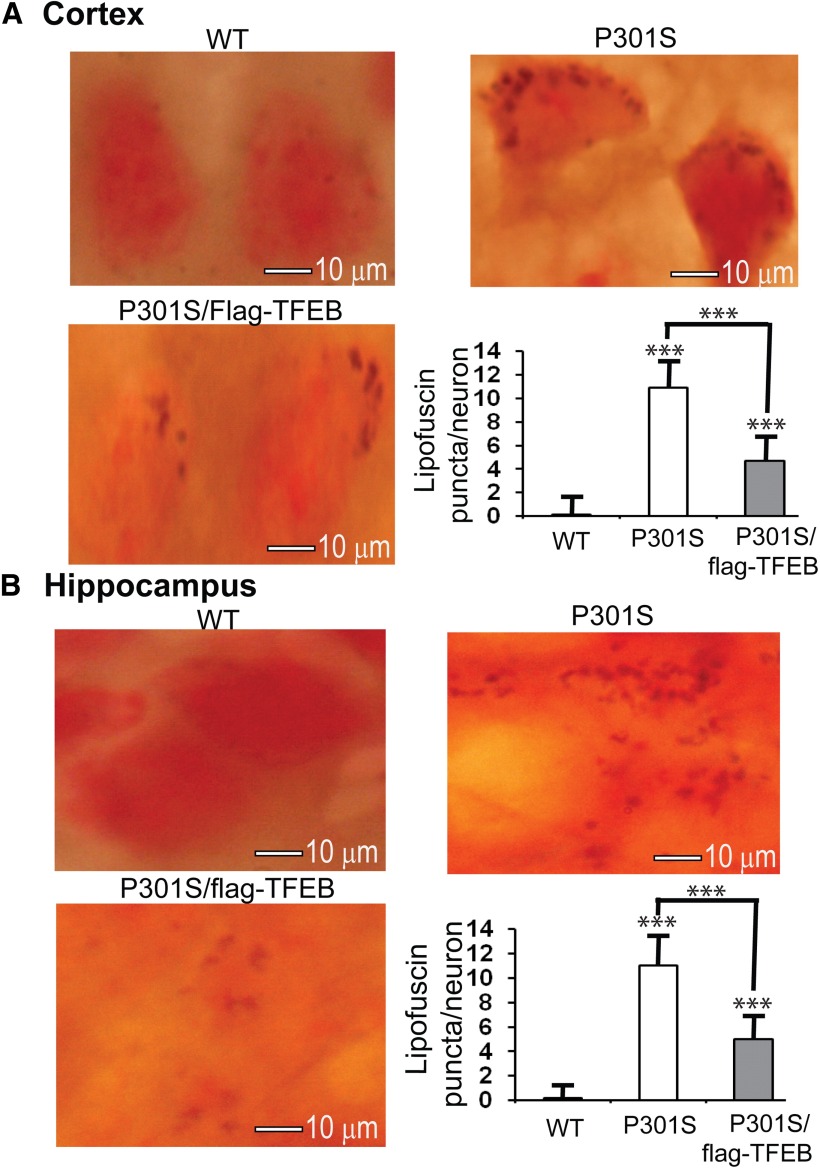
TFEB expression significantly reduces the number of lipofuscin puncta in both the cortex and hippocampus of the P301S model of tauopathy. ***A***, Coronal brain sections from the three genotypes of mice as shown were stained with 1% SBB for 8 min and then counterstained with 0.1% Nuclear Fast Red for 10 min. The quantitation of lipofuscin density in the frontal cortical neurons revealed an average of ∼10.9 granules/neuron in the P301S tauopathy model, which was significantly reduced to 4.6 granules/neuron, a reduction of 57%, in the double-transgenic mice. WT neurons barely showed any lipofuscin granules. ***B***, Brain sections were similarly processed as in ***A***, and lipofuscin granules were quantified in the CA1 region of the hippocampus, which showed an average of 11 granules in the P301S mice and 4.9 in the double-transgenic mice, a reduction of 55% due to TFEB overexpression. Statistical analysis by one-way ANOVA followed by Student–Newman–Keuls *post hoc* test revealed significant differences. ***p < 0.001, compared to WT or P301S mice, as indicated. The data are reported as the mean ± SEM. *n* = 5 per genotype.

After documenting surprising results concerning the role of TFEB in increasing SA-β-gal-positive neurons in the mouse brains, we were interested to examine lipofuscin, another age-associated pigment deposited in the brain. Lipofuscin is a complex and dynamic form of intracellular protein aggregates, composed primarily of protein and lipid, with trace amounts of carbohydrates and metals. As autophagy induction is known to clear intracellular protein aggregates, it was interesting to also study the effect of TFEB on lipofuscin granules in the brain. At 7 months of age, lipofuscin was undetectable in most neurons of both cortical and hippocampal brain regions in the WT mice (Fig. 8*A*,*B*). However, in the P301S model of tauopathy, we found many neurons showing deposits of lipofuscin in the form of dark-colored puncta. In the cortex, the average number of lipofuscin puncta per neuron were found to be ∼10.92 (*p* < 0.001) in the P301S mice compared with <1 in the WT controls. Interestingly, TFEB overexpression in the P301S mice (P301S/flag-TFEB) significantly reduced the number to 4.67 (57%; *p* < 0.001; Fig. 8*A*). In the hippocampus, neurons from WT mice also showed almost no deposition of lipofuscin, while P301S mouse neurons showed an average of 11.03 puncta/neuron, whereas TFEB overexpression reduced it to 4.97 puncta/neuron (55%, *p* < 0.001) in the P301S/flag-TFEB double-transgenic mice (Fig. 8*B*). Thus, although lipofuscin puncta are normally considered nondegradable once they are deposited, overexpression of a transcription factor such as TFEB clearly reduces the lipofuscin deposits in both the cortex and the hippocampus. This observation undoubtedly has tremendous implications for age-associated neurological disorders, wherein lipofuscin deposition has been shown to accelerate neurodegeneration.

### TFEB overexpression significantly prevents learning and memory deficits in the P301S mice

Since hyperphosphorylated tau and synaptic pathology correlate with learning and memory deficits in the P301S model of tauopathy (Xu et al., 2014), and since TFEB overexpression significantly reduces PHF tau levels, we were interested to verify whether TFEB expression also has any impact on behavior. While the T-maze alternation paradigm imposes a more stringent working memory demand on the mice (Stewart et al., 2011), the Barnes maze provides an *in vivo* neurophysiological readout of hippocampal function and permits one to “visualize” the encoding and long-term retention of new spatial memory as the mouse explores a novel environment (Fox et al., 1998). Therefore, we used the Barnes maze as a test of spatial memory, and the T maze alternation task as a test of working and reference memories.

**Figure 9.**
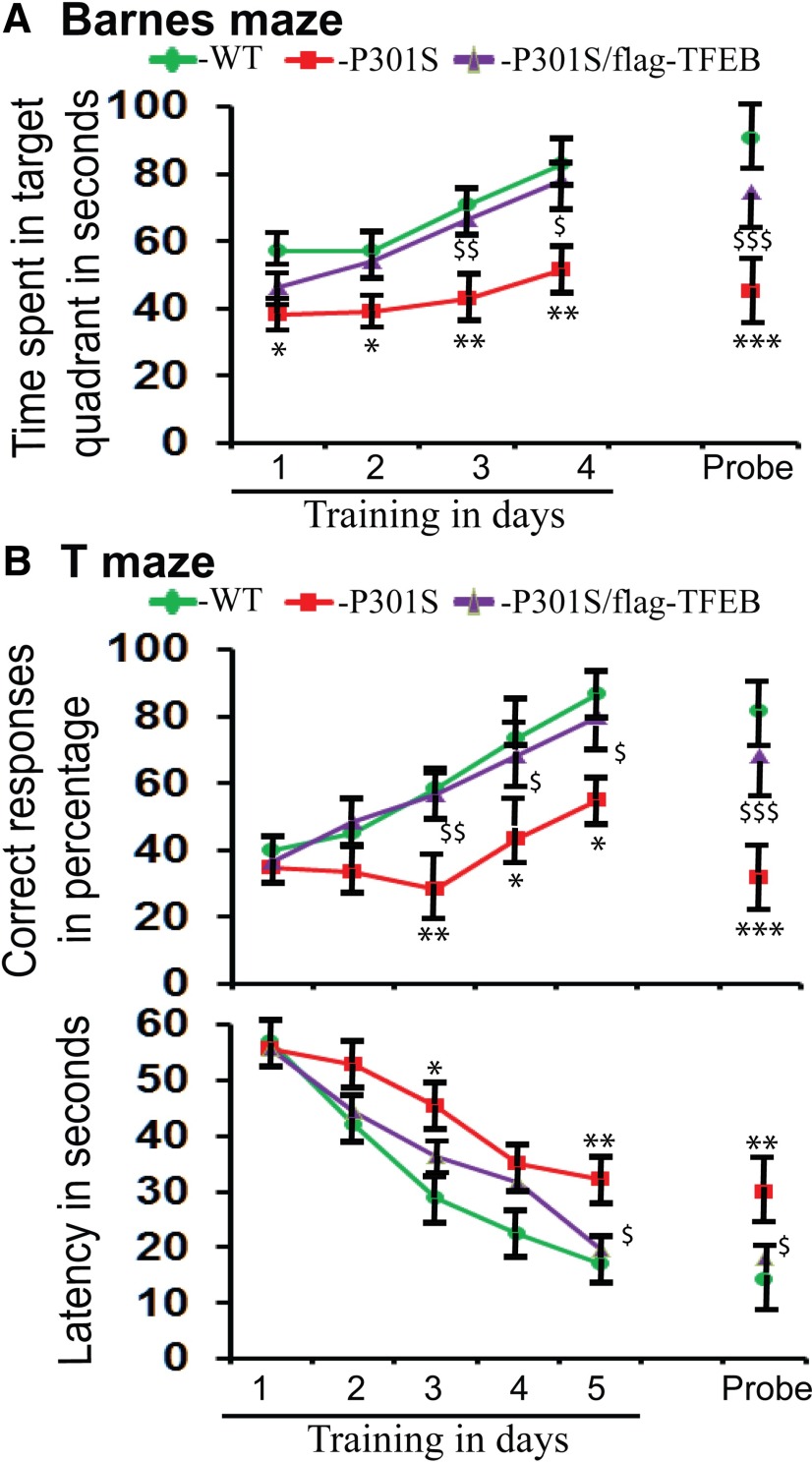
TFEB expression in the P301S mice significantly attenuates learning and memory deficits. ***A***, In the Barnes maze test, on day 1 of training, time spent in the target quadrant was 33% less by P301S mice and only 18% less by P301S/flag-TFEB double-transgenic mice compared with WT controls. On day 2, P301S mice spent 32% less time, but double-transgenic mice spent only 5% less time. On day 4, the difference was 38% for P301S mice and only 6% for the double-transgenic mice. During the probe test, the difference was from 50% to 17%. ***B***, In the T maze paradigm, on day 3 of testing, the correct responses were 58% for WT mice, 28% for P301S mice, and 57% for double-transgenic mice. On day 4 of testing, the correct responses were 73% for WT mice, 43% for P301S mice, and 68% for double-transgenic mice. On day 5, the correct responses were 87% for WT mice, 55% for P301S mice and 80% for the double-transgenic mice. During the probe test, the correct responses were 82% for WT mice, 32% for P301S mice, and 68% for the double-transgenic mice. The latency also differed. On day 3, the average latencies for WT, P301S, and double-transgenic mice were 29, 45, and 36 s, respectively. On day 5, the average latencies for WT, P301S, and double-transgenic mice were 17, 32, and 19 s, respectively. On the day of the probe test, the latencies for WT, P301S, and double-transgenic mice were 14, 30, and 18 s, respectively. *p < 0.05, **p < 0.01, and ***p < 0.001, compared with WT controls. $$*p* < 0.01, $$$*p* < 0.001, compared with P301S mice. The data are reported as the mean ± SEM. *n* = 6 per genotype.

In the Barnes maze test, P301S mice showed significant differences in terms of time spent in the target quadrant both during the training period and the probe test, which were significantly attenuated when TFEB was overexpressed in the P301S/flag-TFEB double-transgenic mice (Fig. 9*A*). On day 1 of training, P301S mice spent 33% (*p* < 0.05) less time compared with WT mice, but in the double-transgenic mice the difference was only 18%. On day 2, the difference was 32% (*p* < 0.05), which was attenuated to 5% in the double-transgenic mice. On day 3, the difference was 39% (*p* < 0.01), attenuated to 6% (*p* < 0.01), and similarly on day 4, the difference was 38% (*p* < 0.01), attenuated to 6% (*p* < 0.01) in the double-transgenic mice (Fig. 9*A*). During the probe test, P301S mice spent 50% (*p* < 0.001) less time compared with WT mice, which was attenuated to 17% (*p* < 0.001) in the double-transgenic mice (Fig. 9*A*). Thus, P301S mice showed significant deficits in both learning and memory tasks in the spatial memory test. Importantly, TFEB overexpression significantly attenuated such a deficit.

In the T maze test, P301S mice performed similarly to WT mice during days 1 and 2 of training (Fig. 9*B*). However, on day 3, WT mice scored 58% correct responses, but P301S mice scored only 28% (*p* < 0.01), which was significantly reversed to 57% (*p* < 0.01) in the double-transgenic mice. On day 4, WT mice scored 73% correct responses, but P301S mice scored only 43% (*p* < 0.05), which was significantly reversed to 68% (*p* < 0.05) in the double-transgenic mice due to TFEB overexpression. On day 5, correct responses were 87% for WT mice, 55% (*p* < 0.05) for P301S mice, and 80% (*p* < 0.05) for the double-transgenic mice (Fig. 9*B*). During the probe test, WT mice showed 82% correct responses, while P301S mice showed 32% (*p* < 0.001) and double-transgenic mice showed 68% (*p* < 0.001; Fig. 9*B*). In terms of latency, P301S mice did not show any difference on days 1, 2, and 4. On day 3 of training, the average latency for WT mice was 29 s, for P301S mice was 45 s (*p* < 0.05), and for double-transgenic mice was 36 s. On day 5, the latencies were 17 s for WT mice, 32 s (*p* < 0.01) for P301S mice, and 19 s (*p* < 0.05) for the double-transgenic mice (Fig. 9*B*). During the probe test, the latencies were 14 s for WT mice, 30 s for P301S mice (*p* < 0.01), and 18 s for the double-transgenic mice (*p* < 0.05; Fig. 9*B*). Thus, P301S mice showed deficits in both correct responses and the latency compared with WT mice, which was significantly attenuated by TFEB expression.

## Discussion

In this study, we made several novel observations. First, we demonstrated that bicistronic vectors can be used to express two proteins in the transgenic mouse brains safely and efficiently by taking advantage of the self-processing property of the viral 2A peptide. Second, although surprising, TFEB overexpression increases rather than decreases the SA-β-gal activity in the mouse brain. Third, and most importantly, TFEB overexpression reduces PHF-tau levels in the cortical and hippocampal brain regions of the P301S model of tauopathy. Finally, and most remarkably, TFEB overexpression reduces the number of lipofuscin puncta *in vivo* in the mouse brains. Thus, the TFEB overexpression strategy is likely to have therapeutic potential for tauopathies, including AD, which are characterized by the deposition of protein aggregates partly due to impaired autophagy.

SA-β-gal activity has been most extensively used as a biomarker of senescent cells ever since it was described ∼2 decades ago (Dimri et al., 1995). Based on the evidence that enhanced autophagy prolongs lifespan in several organisms (Simonsen et al., 2008; Eisenberg et al., 2009; Morselli et al., 2010), including mice (Pyo et al., 2013), our idea of quantifying SA-β-gal-positive neurons was based primarily on the presumption that TFEB overexpression/autophagy induction might reduce the number of age-associated SA-β-gal-positive neurons in the mouse brains. But, contrary to our prediction, we were initially quite puzzled to explain the increased number of SA-β-gal-positive neurons. In recent years, the suitability of SA-β-gal as a marker of senescent cells has been questioned (Cristofalo, 2005; Yang and Hu, 2005). In these studies, it was found that SA-β-gal activity was increased not only with cell aging, but also in nonsenescent states such as confluent quiescent cells or cells deprived of serum. Because the SA-β-gal activity could subsequently be attributed to the lysosomal β-galactosidase activity, these studies concluded that SA-β-gal activity is nonspecific to senescence. Indeed, using a genetic approach, Lee et al. (2006) finally confirmed that SA-β-gal activity is encoded by the lysosomal β-galactosidase gene GLB1 and provided indisputable evidence that the increased SA-β-gal activity observed previously in various senescent cells is due only to increased lysosome content. Thus, SA-β-gal activity does not reflect the senescence state, but rather indicates only increased lysosomal activity in the senescent cells. Our results are consistent with this idea. TFEB, being a transcription factor known to induce lysosome biogenesis (Sardiello et al., 2009), is expected to enhance SA-β-gal activity due to increased lysosome numbers. Thus, the robust increase in the number of neurons with SA-β-gal activity in the cortex and hippocampus observed in the present study indirectly reflects increased lysosome numbers due to TFEB overexpression in these brain regions. Compelling direct evidence for the increased number of lysosomes also comes from the increased protein levels of lysosomal membrane marker LAMP1. Increased protein levels of cathepsin D is additional compelling evidence to support increased lysosomal activity in the transgenic mice overexpressing flag-TFEB. Indeed, ultrastructural analysis of these brain regions by TEM confirmed increased lysosome numbers in the mice overexpressing flag-TFEB. These multiple pieces of evidence clearly suggest that TFEB overexpression, consistent with previous observations in cell cultures, stimulates lysosome activity and that increased SA-β-gal activity per se just reflects that activity, and not senescence.

Another important finding in the present study is the reduced levels of PHF-tau, but not of total tau, in the P301S mouse model of tauopathy due to TFEB overexpression-mediated autophagy. These results are consistent with another recent study by Polito et al. (2014), which also demonstrated similar effects on PHF-tau in the rTg4510 model of tauopathy carrying the P301L mutation. Our study, together with the study by Polito et al. (2014), strongly suggests that TFEB can rescue paired helical filaments caused by different MAPT mutations. While we used a conventional transgenic model and overexpressed human TFEB throughout the brain, Polito et al. (2014) used adeno-associated virus to express mouse TFEB and directly injected the mouse cDNA into the brain lateral ventricles of day 1 postnatal pups. Thus, TFEBs from both humans and mice are effective in reducing PHF-tau levels. These results are in agreement with previous demonstrations that autophagy activation by rapamycin (Ozcelik et al., 2013), trehalose (Schaeffer et al., 2012), and methylene blue (Congdon et al., 2012) reduces PHF-tau levels *in vivo*, while autophagy inhibitors, such as chloroquine, NH4Cl, 3-methyladenine, and cathepsin inhibitors, enhance the levels of PHF-tau. More conclusive evidence that basal autophagy is essential for the degradation of phosphorylated tau comes from studies in conditional Atg7 knock-out mice, which showed enhanced tau phosphorylation. Interestingly, the inhibition of tau phosphorylation by either a pharmacological or genetic approach in the background of Atg7 deficiency significantly reduced neurodegeneration (Inoue et al., 2012), suggesting that basal autophagy is essential to prevent tau phosphorylation as well as its aggregation-mediated neurodegeneration.

The mechanism by which TFEB lowered the levels of PHF-tau appears to be due to increased cathepsin D-mediated degradation. Previous studies (Bednarski and Lynch, 1996; Kenessey et al., 1997) have shown that tau can be degraded by cathepsin D *in vitro* and *in vivo*. Our observation that TFEB overexpression increases cathepsin D levels in both hippocampus and cortex is also consistent with observations from a previous study (Xiao et al., 2014). Moreover, cathepsin D protein levels are reduced in the human AD brain (Urbanelli et al., 2008), and polymorphisms in the cathepsin D gene increases AD risk (Papassotiropoulos et al., 2000; Beyer et al., 2005; Schuur et al., 2011). These pieces of evidence, when taken together with the present results, suggest that increased cathepsin D by TFEB overexpression may be responsible for clearing PHF-tau in the P301S mouse brains. However, it should be noted that both the inhibition of cathepsin D by a potent cathepsin D inhibitor in cultured hippocampal slices (Bi et al., 2000) and decreased cathepsin D protein levels due to immunization with phosphorylated tau peptides in the mouse brain (Boimel et al., 2010) have been shown to reduce pathological tau species. There are also reports that cathepsin D does not degrade pathological tau (Wang et al., 2009). Although these pieces of evidence suggest a dual role for cathepsin D in tau degradation, since genetic ablation of cathepsin D significantly increases tau-induced neurotoxicity (Khurana et al., 2010), it is very likely that increased cathepsin D is responsible for the reduced levels of PHF-tau. The reduced PHF-tau level in turn is likely to prevent synaptic loss and neurodegeneration, as reflected by increased levels of synaptic proteins such as spinophilin and the neuronal marker NeuN, in the double-transgenic mice.

The most remarkable finding in the present study is the reduction of lipofuscin puncta in the double-transgenic mice overexpressing mutant tau and flag-TFEB. Lipofuscins may contain undigested membranes, and aggregates of misfolded proteins, lipids, and metals. Our finding is important because, once formed in the neurons, lipofuscin granules are generally considered to be nondegradable. Also, although lipofuscins are deposited consistently in very old mice, P301S mouse brains showed lipofuscin deposits even by 7 months of age. In AD patient brains, those neurons bearing tau tangles also show significantly elevated levels of lipofuscin (Sumpter et al., 1986), which supports the present findings. Lipofuscin deposition is presumed to result from reduced lysosomal hydrolytic capacity as varieties of lysosomal inhibitors have been shown to induce lipofuscins (Terman et al., 1999; Szweda et al., 2003). Since TFEB overexpression induces lysosome biogenesis, the resulting increased hydrolytic capacity of lysosomes, including increased cathepsin D levels, could account for the reduced numbers of lipofuscin puncta. A direct and critical role for cathepsin D in the metabolism of lipofuscins comes from cathepsin D-deficient mice, which showed increased deposition of one type of neuronal ceroid lipofuscinosis (Koike et al., 2000). Moreover, in human patients a complete loss of cathepsin D results in a devastating form of the lysosomal neurodegenerative disorder called ceroid lipofuscinosis (Siintola et al., 2006; Steinfeld et al., 2006; Jalanko and Braulke, 2009). This suggests that an increase in cathepsin D levels by TFEB is also responsible for reducing the number of lipofuscin puncta. Additionally, TFEB can also increase the exocytosis of lysosomes through the plasma membranes (Medina et al., 2011), thereby excluding aggregated and nondegradable materials such as lipofuscins from neuronal cytosol. Our results on lipofuscin are also in agreement with the published reports that TFEB overexpression could promote the clearance of lipofuscins from patient-derived fibroblasts (Medina et al., 2011). Thus, contrary to the long-held view that lipofuscins are nondegradable, TFEB overexpression indeed can, to some extent, reverse the accumulation of lipofuscin puncta from neurons. This finding undoubtedly has enormous clinical implications for multiple pathologies.

Another important finding in the present study is the marked attenuation of cognitive deficits seen in the P301S mice when TFEB was overexpressed. The trend toward normalization of cognitive deficits due to TFEB expression is comparable between T maze and Barnes maze paradigms, suggesting consistency in the effects of TFEB. The observed deficits in learning and memory skills in the P301S mice are also consistent with previous results demonstrated by different paradigms, including Barnes maze (Takeuchi et al., 2011), Y maze (Takeuchi et al., 2011), and Morris water maze (Xu et al., 2014; Jiang et al., 2015). Double-mutant transgenic mice expressing both P301S and K257T mutations under the natural tau promoter also showed severe deficits in spatial memory, as assessed by T maze and eight-arm mazes, and were also accompanied by deficits in long-term potentiation (LTP) *in vivo* (Rosenmann et al., 2008). How could TFEB expression ameliorate learning deficits? Since LTP is considered to be a cellular basis for learning and memory, and also since hyperphosphorylated tau correlates with learning deficits (Joshi et al., 2012; Jiang et al., 2015), reduction of PHF-tau by TFEB expression is likely responsible for the reversal of memory deficits. Reduced PHF-tau levels in turn could reduce neurodegeneration and synaptic loss, as reflected by the restoration of NeuN and spinophilin levels, especially in the cortex and hippocampus. Since these two brain regions are crucial for maintaining normal cognition levels (Burgess et al., 2002; Donoso et al., 2014), the restoration of neurodegenerative and synaptic pathology in these regions could lead to behavioral recovery. However, it should be noted that in the P301S mice learning and memory deficits are seen as early as 2.5 months of age (Xu et al., 2014), when these mice do not display PHF-tau, tangle pathology, or overt neurodegeneration (Yoshiyama et al. 2007). Instead, a remarkable loss of synapses and impaired synaptic function are detectable as early as 3 months of age in the hippocampus of P301S mice, suggesting that the loss of synaptic integrity is likely the cause of behavioral deficits. At this age, these mice also show an accumulation of tau-positive spheroids due to defects in axonal transport (Yoshiyama et al., 2007). It is important to note that defects in axonal transport lead to autophagic impairment and subsequent clearance deficits for aggregated proteins (Ravikumar et al., 2005). Thus, autophagy defects through axonal transport impairment lead to axonal dystrophy, which in turn could lead to axonal degeneration. TFEB-mediated enhancement of the entire autophagy pathway (Sardiello et al., 2009; Settembre et al., 2011) could theoretically prevent axonal transport defects and, therefore, axonal degeneration. The fact that autophagy activators such as rapamycin (Ozcelik et al., 2013) and trehalose (Schaeffer and Goedert, 2012) significantly ameliorate neuropathology in the P301S mice supports our interpretation.

In conclusion, these findings suggest that TFEB activation stimulates the autophagy–lysosome pathway, resulting in the clearance of PHF-tau and lipofuscins, which in turn may rescue loss of synapses, and learning and memory deficits. It is also possible that reduced PHF-tau levels and cognitive deficits may be mediated through two parallel signaling pathways and may not have a causal relationship. In the absence of disease-modifying therapy, a small-molecule activator of TFEB is therefore expected to have therapeutic potential for the treatment of AD, other tauopathies, as well as several other neurological disorders characterized by the accumulation of protein aggregates.

## Synthesis

The decision was a result of the Reviewing Editor Alfonso Araque and the peer reviewers coming together and discussing their recommendations until a consensus was reached. A fact-based synthesis statement explaining their decision and outlining what is needed to prepare a revision is listed below. The following reviewers agreed to reveal their identity: Hanna Rosenmann, Dezhi Liao

I concur with the reviewers' comments expressing the novelty and quality of the study. I believe that addressing the minor comments expressed will definitely improve the manuscript.

Specific comments of the reviewers:

Reviewer 1.

This is an excellent high quality paper manuscript. A genetic model overexpression of TFEB was generated and systematically characterized to show that can reliably enhance autophagy in vivo. Using this new model it was shown to reduce levels of PHF-tau, deposition of lipofuscin granules and learning and memory deficits; and may therefore have potential therapeutic impact.

Minor comments:

1. In Discussion: Indeed there is evidence that cathepsin D reduces pathogenic tau, however there is also another mechanism by which inhibiting cathepsin D reduces pathogenic tau (Xiaoning Bi, J Neuroch 2010; Boimel M, Exp Neurol 2010; Wang Y, Hum Mol Genet 2009).

2. In Discussion: other models for P301S showing learning deficits, including LTP deficits (Rosenmann H, Exp Neurol 2008).

3. In Abstract: to add the important results of response of the synaptic markers.

Reviewer 2.

The present manuscript describes the creation and characterization of new transgenic mouse lines that co-express TFEB and EYFP, which are inserted into bicistronic vectors. Due to the recent publication of a similar paper by Hui Zheng's group (Polito et al., 2014, EMBO), the present paper provides little conceptual advance for the field per se. However, the approaches used here are very different from the previous study (transgenic mice here; viral expression in Polito's paper). The reviewer believe that studies using transgenic mice can rule out some of non-specific effects of viral studies arising from variation of protein expression levels. Therefore, even though the two papers reach almost the same conclusions, due to the extraordinarily high significance of the finding that TFEB can clear up PHF tau for tauopathies, the present paper will, without doubt, provides advances in the field. Moreover, the creation of the novel mouse lines will allow live imaging studies of synaptic structures due to the simultaneous presence of EYFP in the neuron, which will be very useful for future mechanistic studies. The reviewer has two minor points:

1. The conclusion needs to be softened. The causal relationship between PHF clearance and cognitive deficits has not been proven in the mostly correlative studies here. The above two changes may be mediated through two parallel signaling pathways that depend upon TFEB.

2. It is unclear to the reviewer whether TFEB can change the morphology of dendritic spines. As the neurons express EYFP, some live imaging studies of dendritic spines in neurons cultured from these transgenic mice will significantly increase the impact of the present study.
